# Supplementary material for: Development of an easy-to-use questionnaire assessing critical care nursing competence in Japan: A cross-sectional study
Source: PLoS One. 2019 Nov 27;14(11):e0225668. doi: 10.1371/journal.pone.0225668 (PMC6881035; doi:10.1371/journal.pone.0225668)
Supplement: Attachment — Submitted filename: Response to Reviewers.docx [file pone.0225668.s002.docx]

PONE-D-19-19921

Development of an easy-to-use questionnaire assessing critical care nursing competence in Japan: a cross-sectional study

Dear Dr. Soundy:

Thank you for your ongoing consideration of our manuscript (PONE-D-19-19921) for publication in the PLOS ONE. We appreciate the time spent by you and the reviewer and believe the revised manuscript is improved. Below, we address the reviewers’ comments.

We look forward to hearing from you regarding our submission. We would be glad to respond to any further questions and comments that you may have.

Sincerely,

Masatoshi Okumura, MSc, MD

Department of Anesthesiology, Aichi Medical University,

1-1 Yazakokarimata, Nagakute, Aichi 480-1195, Japan

Tel: +81-561-62-3311	Fax: +81-561-62-4866

E-mail: okuchin0518er@gmail.com

-----

JOURNAL REQUIREMENTS:

1. **When submitting your revision, we need you to address these additional requirements. Please ensure that your manuscript meets PLOS ONE's style requirements, including those for file naming.**

**Response:** In accordance with the Journal Requirements, we have corrected our manuscript to PLOS ONE's style.

2. **We note that you have included the phrase “data not shown” in your manuscript. Unfortunately, this does not meet our data sharing requirements. PLOS does not permit references to inaccessible data. We require that authors provide all relevant data within the paper, Supporting Information files, or in an acceptable, public repository. Please add a citation to support this phrase or upload the data that corresponds with these findings to a stable repository (such as Figshare or Dryad) and provide and URLs, DOIs, or accession numbers that may be used to access these data. Or, if the data are not a core part of the research being presented in your study, we ask that you remove the phrase that refers to these data.**

**Response:** In accordance with the Journal Requirements, we have changed the following text

from

(Lines 225-229)

"ICU nurses with experience in emergency departments also showed significantly higher scores than did those without this experience (p < 0.001), but there was no significant difference in these scores between ICU nurses with and without experience in cardiovascular departments or operating rooms (data not shown)."

to

(Lines 228-233)

"ICU nurses with experience in emergency departments also showed significantly higher scores than did those without this experience (16.7 [SD 1.48] vs. 15.7 [SD 1.67], respectively, p < 0.001), but there was no significant difference in these scores between ICU nurses with and without experience in cardiovascular departments (16.4 [SD 1.80] vs. 15.9 [SD 1.64], respectively, p 0.08) or operating rooms (16.1 [SD 1.67] vs. 16.0 [SD 1.67], p 0.84)."

-----

RESPONSE TO REVIEWER:

**Comment 1**: Introduction

**Is the number of the certified nurses (in intensive care) correct ? According to the reference, it is described as 1033. The CNS (critical care nursing) is not mentioned throughout in this paper. I think the certified nurse specialists work at ICU, but why is it excluded?**

**Response:**

We appreciate these helpful suggestions. We have written the latest number of nurses that have not yet been translated into English. To avoid confusion, we have changed the numbers as referenced.

Next, we didn't mention CNS, because CNS is small and rarely seen in the clinical practice. However, in our questionnaire C3Q-safety, we did not distinguish between CN and CNS. Therefore, CNS may be included in the CN group. As you indicated, we have changed it to the correct text.

From

(Lines 68-70)

"In terms of the medical environment in Japan, as of 2017, there were 1,169 certified nurses in intensive care among 805,708 full-time registered nurses (RNs) in the country."

To

(Lines 66-68)

"In terms of the medical environment in Japan, as of 2016, there were 1,033 certified nurses (CNs) in intensive care and 177 certified nurse specialists (CNSs) in critical care nursing among 805,708 full-time registered nurses (RNs) in the country."

Further, we have added a new sentence.

(Lines 75-77)

"To become a CNS in critical care nursing, an RNs with more than three years of experience in a specialized field must complete a master's course at a graduate school and then pass an examination administered by the JNA."

Finally, we have rewritten from CN to CN/CNS in several points.

**Comment 2:** Material and Methods

**In the process of preparing the questionnaire, verification of reliability and validity is necessary. I think stability verification is necessary, because this questionnaire assesses competency. If not, I think you should add to the limits of the research.**

**What is the cut point of factor loading in exploratory factor analysis? The factor loading of item No, 5, 11, 15, and 23 seem to be low. Why these items are adapted?**

**There is no description of the results of confirmatory factor analysis (model fit or coefficient). If verified, description is required.**

**Response:**

We appreciate your suggestions. As suggested, we have added the following sentence in reliability and validity.

(Lines 316-329)

"Third, we should mention limitation in reliability and validity of the C3Q-safety. Internal consistency reliability of the C3Q-safety was acceptable, because Cronbach's alpha coefficients varied between 0.73 and 0.83. Cronbach’s alpha coefficient greater than 0.7 was considered to indicate acceptable reliability. However, the instrument's repeatability and reliability have not been assessed by test-retest analysis. Regarding the validity, we also need further verification. The C3Q-safety is composed of questions that are easy to understand, small in number, and well-reviewed by critical care experts, therefore, the content validity could be maintained to some extent. Although we have not assessed the external validity of the C3Q-safety using previous measurement scales, a higher score of the C3Q-safety depending on critical care certification and years of nursing experience is considered to indicate a certain degree of the external validity. In the future, the comparison of previous measurement scales with the C3Q-safety will reveal the C3Q-safety's characteristics and then lead to using these scales for different purposes in accordance to the goal."

As you pointed out, factor loading of No 5, 11, 15, and 23 is low. In fact, we already analyzed after excluding these items with some combinations. As a result, the exclusions have changed the combination of items that construct the four factors, and have led to a decrease in Cronbach's alpha coefficient. Furthermore, the changed structure was different from the ideal structure we assumed. Another reason is that these low factor loading items are good questions. Considering all, we decided on this factor structure.

Confirmatory factor analysis is not performed in this paper. We plan to report it in the following study. We have removed description of "confirmatory factor analysis" from the Materials and Methods.

**Comment 3**: Discussion

**The competence is compared by multiple back grounds of nurses. Certified nurses are included in comparison of years of experience. The years of experience of certified nurses are unknown. I can’t recognize whether the influence of competence is due to a certified license or years of experience.**

Response:

We agree with your assessment. As suggested, from this study, we could not distinguish the effects of license and years of experience on CN/CNS's higher competence. We have discussed this issue in the Limitations section (Lines 310-315).

Thank you again for your comments on our paper. I hope that the revised manuscript would be now suitable for the publication.
